# Strategies to improve global influenza surveillance: A decision tool for policymakers

**DOI:** 10.1186/1471-2458-8-186

**Published:** 2008-05-28

**Authors:** Melinda Moore, Edward Chan, Nicole Lurie, Agnes Gereben Schaefer, Danielle M Varda, John A Zambrano

**Affiliations:** 1 Health Unit, RAND Corporation, Arlington, Virginia, USA; 2 Health Unit, RAND Corporation, Santa Monica, California, USA; 3 Health Unit, RAND Corporation, Pittsburgh, Pennsylvania, USA

## Abstract

**Background:**

Global pandemic influenza preparedness relies heavily on public health surveillance, but it is unclear that current surveillance fully meets pandemic preparedness needs.

**Methods:**

We first developed a conceptual framework to help systematically identify strategies to improve the detection of an early case or cluster of novel human influenza disease during the pre-pandemic period. We then developed a process model (flow diagram) depicting nine major pathways through which a case in the community could be detected and confirmed, and mapped the improvement strategies onto this model. Finally, we developed an interactive decision tool by building quantitative measures of probability and time into each step of the process model and programming it to calculate the net probability and time required for case detection through each detection pathway. Input values for each step can be varied by users to assess the effects of different improvement strategies, alone or in combination. We illustrate application of the tool using hypothetical input data reflecting baseline and 12-month follow-up scenarios, following concurrent implementation of multiple improvement strategies.

**Results:**

We compared outputs from the tool across detection pathways and across time, at baseline and 12-month follow up. The process model and outputs from the tool suggest that traditional efforts to build epidemiology and laboratory capacity are efficient strategies, as are more focused strategies within these, such as targeted laboratory testing; expedited specimen transport; use of technologies to streamline data flow; and improved reporting compliance. Other promising strategies stem from community detection – better harnessing of electronic data mining and establishment of community-based monitoring.

**Conclusion:**

Our practical tool allows policymakers to use their own realistic baseline values and program projections to assess the relative impact of different interventions to improve the probability and timeliness of detecting early human cases or clusters caused by a novel influenza virus, a possible harbinger of a new pandemic. Policymakers can use results to target investments to improve their surveillance infrastructure. Multi-national planners can also use the tool to help guide directions in surveillance system improvements more globally. Finally, our systematic approach can also be tailored to help improve surveillance for other diseases.

## Background

As the world confronts the extensive spread of influenza A/H5N1 virus among animals and the growing number of cases in humans, it has an unprecedented opportunity to prepare for the next human influenza pandemic. Many experts feel that the world is overdue for such a pandemic. Since the pandemics of the past century (1918, 1957, 1968), the world has become more globalized in terms of travel and trade, thus facilitating greater extent and speed of disease spread. At the same time, however, the world has also become more experienced in addressing emerging infectious disease threats.

Among the many priority activities related to pandemic preparedness, most experts would agree on the critical role played by public health surveillance, defined as "the ongoing systematic collection, analysis, interpretation and dissemination of data regarding a health-related event for use in public health action to reduce morbidity and mortality and to improve health" [1], or more simply: systematic information for public health action. The Institute of Medicine has noted that "The importance of surveillance to the detection and control of emerging microbial threats cannot be overemphasized" [2].

In the context of pandemic preparedness, surveillance is critical for the early detection and timely response to contain or limit the spread of novel human influenza viruses to which humans have no immunity, such as A/H5N1 or other strains that may yet emerge. Global preparedness is strengthened when surveillance systems in all countries are capable of detecting early cases of novel human influenza within their borders. Pandemic preparedness guidance from the World Health Organization (WHO) [3] and the U.S. Government's recent *National Strategy for Influenza Preparedness – Implementation Plan *[4] both include prominent attention to global surveillance and detection. The WHO Global Influenza Surveillance Network is a longstanding surveillance program whose traditional focus has been the monitoring of influenza virus strains, principally for purposes of determining the appropriate formulation for each season's influenza vaccine. However, it is unclear how well this system would serve the need for reliable early detection of novel influenza cases that might be a harbinger of an emerging pandemic [5-7]. Therefore, it makes sense to examine possible ways to improve global influenza surveillance, specifically pre-pandemic surveillance to detect early human cases or clusters caused by a novel influenza virus wherever they may occur, before the disease spreads and triggers a pandemic. Moreover, approaches to improve global influenza surveillance could also have implications for surveillance and early detection of other diseases.

This study addresses two questions: What are the key elements of influenza surveillance? How might investments in them lead to improvements in reliable and early detection? In this paper, we describe a quantitative decision tool that we developed to help policymakers assess the relative effects of improving different elements of their influenza surveillance system. The tool is based on a process model (or flow diagram) that depicts the nine major pathways through which a novel influenza case could be detected and confirmed. We identified potential improvement strategies, mapped these onto the relevant steps in the model, built in quantitative input measures of probability and time that users can vary for each step depending on the strategy chosen, and programmed the model to calculate the net probability and time required for each detection pathway. This allows policymakers to use the tool to compare the probability and timeliness of detection for each of the detections pathways, assess the implications of specific elements of their surveillance system, and project realistic improvements over time. The intent is to help the user identify targeted investments to improve surveillance. To help extend the reach of internal efforts to improve surveillance systems, we also describe opportunities for policymakers to leverage partners to help improve their surveillance coverage, quality and timeliness. Using these approaches – the decision tool and opportunities to leverage partners – we identify a number of practical ways to help improve global influenza surveillance. While the work described here was conducted for the U.S. Department of Health and Human Services to help guide its global influenza surveillance programming, our goal was to develop a tool that is broadly applicable around the world.

## Methods

To develop a conceptual framework that could facilitate the identification and organization of potential improvements in global influenza surveillance, we first reviewed guidance published by the World Health Organization (WHO) and the U.S. Centers for Disease Control and Prevention to glean criteria for evaluating public health surveillance systems and to create a simplified framework representing a core set of surveillance requirements. We then sought and reviewed published reports from anywhere in the world for evidence or suggestions regarding improvements to influenza surveillance. We searched the MEDLINE database using such search terms as "influenza", "surveillance", "early", "warning", and "detection", alone or in combination. Based on review of reports identified through this search, we identified and organized improvement strategies into a conceptual framework according to three core attributes: surveillance coverage, quality and timeliness.

Using process mapping, a method derived from work in engineering, we next developed a process model (or flow diagram) to show nine different "detection paths", and the steps for each, through which a case in the community could be detected and confirmed. We mapped all of the improvement strategies from the conceptual framework onto the process model. We then developed an interactive tool by building in quantitative measures of probability and time to each step of the process model and programming it to calculate the net probability and total time for each of the nine detection paths. The tool allows users to input their own values for various program elements (corresponding to steps in the model) and see the resulting impact on the likelihood and timeliness of detection – via each different detection path – of a confirmed case of influenza caused by a novel virus strain in the community. Because there is not sufficient evidence on which to base estimates of the performance of the detection paths as they currently exist in most countries, we use a set of plausible hypothetical estimates, including documented planning assumptions when available, to illustrate the use of the tool.

## Results

### Conceptual framework to organize potential improvement strategies

Based on our review of the literature and guidance documents [3,8-10], we identified three core surveillance system attributes that served as the basis for identifying and organizing potential improvement strategies:

• Coverage – includes a broad range of reporting sources and the range of information reported

• Quality – requires accurate information based on standards, trained personnel and quality-assured laboratory testing

• Timeliness – includes rapid detection methods, data flow, analysis, and dissemination to trigger a timely investigation and response to limit or delay disease spread

From our review of published reports, we also identified potential strategies to improve surveillance in these core areas. We identified thirteen strategies in all: four to improve surveillance coverage, three to improve quality and six to improve timeliness (Table 1):

**Table 1:** Thirteen strategies to improve influenza surveillance

*Description*
**COVERAGE: Seek international cooperation and comprehensive surveillance**

(1) **Increase the number/density of traditional reporting sources**
*Increasing the number of traditional reporting sources, e.g., doctors, clinics, hospitals, would increase the percentage of the population covered through surveillance.*
(2) **Develop community-based alert and response systems**
*Community-based surveillance systems would help increase coverage by capturing information on illnesses within the community that may not otherwise reach government attention.*
(3) **Incorporate new human disease sources and signals**
*Gaps in surveillance coverage could potentially be addressed through reporting from new sources (e.g., workplaces, schools, local media, web logs) and new signals (e.g., work or school absenteeism, rumors of compatible cases or outbreaks, or local reports of surge in hospital demand).*
(4) **Increase reporting compliance**
*Assuring the regular voluntary reporting of cases by various surveillance sources (doctors, hospitals, communities, businesses, etc.) assures that the surveillance system in place captures information from all designated reporting sites, thereby improving coverage.*

**QUALITY: Build capacity for accurate, actionable information**

(5) **Improve human laboratory sample preparation and diagnostic capacity**
*Laboratory diagnosis confirms the occurrence of influenza disease and is an important element of surveillance quality; this strategy involves the development and deployment of standard laboratory protocols, training, and regular proficiency testing.*
(6) **Implement targeted laboratory testing appropriate to the pandemic phase and location**
*Potentially scarce laboratory resources can better assure quality services when they are not overwhelmed by demand; criteria for targeted testing, e.g., clinically compatible cases with specified epidemiologic risk factors, should be widely disseminated to and applied by clinical providers.*
(7) **Improve epidemiologic capacity**
*Training in surveillance and applied epidemiology will help improve surveillance quality, including analysis and interpretation of surveillance data and investigation of suspicious cases.*

**TIMELINESS: Ensure rapid case detection, reporting and response**

(8) **Use data mining methods for early warning**
*Capitalizing on data mining methods, such as the Global Public Health Intelligence Network (GPHIN), which employs data mining techniques to systematically scour the electronic news media in multiple languages worldwide for reports of disease occurrences and then process to help discern "signal" from "noise" (14), can improve the timeliness of influenza surveillance.*
(9) **Expand expedited transport of specimens to in-country and international reference laboratories**
*Efforts must be scaled up to assure that specimens anywhere in the world can reach an international laboratory in timely fashion; rapid transport of laboratory specimens from their point of origin (the sick individual) to the first and any subsequent in-country laboratories is also critical.*
(10) **Streamline notification, analysis and reporting**
*Transmission of surveillance data between sub-national and national levels should employ the efficient modalities and channels; reporting electronically or even by telephone should be the desired global norm for surveillance of diseases for which timeliness is a particular priority, including human cases of avian influenza, and electronic technologies should be harnessed for streamlined data processing and analysis.*
(11) **Implement active surveillance when appropriate**
*While active surveillance, in which government authorities directly solicit information about disease occurrence from potential sources such as hospitals and clinical providers, is more labor intensive than routine approaches in which such providers submit reports at regular intervals, selective use of active surveillance can be critical to enhance timeliness of case detection.*
(12) **Develop and deploy rapid laboratory diagnostics with greater sensitivity and reproducibility**
*The development and widespread deployment of more accurate rapid diagnostic tests, including tests that could identify influenza virus subtype, could improve the timeliness of influenza surveillance.*
(13) **Develop and deploy in-country and international Rapid Response Teams to investigate cases/outbreaks**
*The development and deployment of in-country and international Rapid Response Teams can improve the timeliness of surveillance by triggering rapid investigation and subsequent control measures.*

#### Coverage

Strategies to improve surveillance coverage can include increasing the number of traditional reporting sites such as clinics and hospitals participating in surveillance reporting [11,12], addition of new types of sites or information reported [11,12], and community-based reporting, i.e., not from medical facilities [4,13]; increased reporting compliance can further improve coverage from all reporting sources.

#### Quality

Strategies to improve surveillance quality include broad efforts to improve epidemiology and laboratory capacity for investigation and diagnosis [10,11,14], plus targeted laboratory testing so that potentially scarce laboratory resources are used efficiently [11,12].

#### Timeliness

Strategies to improve the timeliness of surveillance include better use of data mining [12] such as the Global Public Health Intelligence Network or GPHIN which scours electronic media reports for clusters of bird die-offs or human deaths [15], expedited transport of specimens [4], streamlined notification and analysis [10-12], implementation of active surveillance when appropriate [12], wide deployment of more accurate rapid diagnostic tests [10,14,16], and deployment of trained Rapid Response Teams to investigate suspicious cases or clusters [4]

### Process model identifying the paths to detection and confirmation

**Figure 1:**
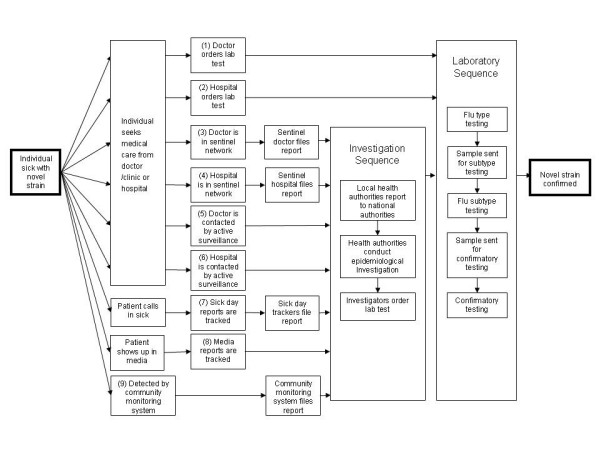
Process model: Pathways and steps toward detection and confirmation of a novel case or cluster of human influenza in the community.

The sequence of steps in the process model is depicted in Figure 1. It begins with a case or cluster of disease in the community caused by a novel influenza virus and ends with detection and laboratory confirmation.

We identified entry points through which a case might be detected, which in turn lead to further steps that must occur so that information is passed along and the case is eventually confirmed. Impediments at any step can mean that detection or confirmation of the case is less likely or delayed. The first six paths begin when a sick individual seeks medical care, through either a doctor/clinic or a hospital; these generally reflect traditional surveillance approaches. The last three paths do not depend on the individual seeking medical care.

Many of these paths lead to an 'Investigation Sequence,' a series of activities that might be triggered by reports of suspicious cases or clusters. For example, the Investigation Sequence may be activated when sub-national health authorities receive reports of influenza-like illnesses from doctors, hospitals, communities or other sources. They, in turn, may notify national health authorities, one or the other of which may begin an epidemiological investigation, which includes the collection of a sample for testing.

All of the paths eventually lead to a 'Laboratory Sequence', a series of steps for testing that ultimately results in laboratory confirmation of a suspicious case. The Laboratory Sequence may be triggered either by the Investigation Sequence, or may be triggered directly by a healthcare provider who orders a laboratory test for an ill individual. In the Laboratory Sequence, the sample is first tested to identify the presence of an influenza virus and possibly the virus type. The sample may be sent for further testing at a more sophisticated laboratory or tested further at the same laboratory.

### A quantitative tool for comparing surveillance strategies

Based on this process model, we developed an interactive decision tool (see Additional file 1). We first built in quantitative measures of probability and time: Each of the steps along the pathways described above has an associated likelihood, or *probability*, of occurring, conditional upon the occurrence of preceding steps. Each step also has an associated *time *required for its completion. At any step, the process can fail to occur altogether or can cause delay in reaching the goal of laboratory confirmation. Achievement at each step, of course, is also dependent on the availability of personnel and resources in a given setting. Therefore, the tool was designed so that users can input their own estimates of the likelihood of occurrence (probability) and time required for completion of each step. Moreover, these values can change over time, e.g., if surveillance programs are improved through implementation of the various improvement strategies. We mapped the 13 surveillance improvement strategies described earlier onto the process model. Each strategy affects one or more steps in the model by increasing the probability that a step occurs and/or decreasing the time required to complete it.

The decision tool allows the user to input quantitative estimates of probability and time for each step in the surveillance process; the tool then calculates the net probability (product of the sequential probabilities in each step along a given path) and total time (arithmetic sum of time required for each step in the path) to reach the final step of laboratory confirmation. Policymakers can apply actual data and realistic projections from their own countries to compare different surveillance strategies and potential improvements to elements of their surveillance systems: They can compare 'baseline' and projected 'follow up' scenarios, i.e., after implementation of selected surveillance improvement strategies. Such quantitative assessment can help policymakers focus their investments on surveillance activities with the greatest promise to improve the probability and timeliness of detection of cases or clusters of disease in the community caused by a novel influenza virus.

### An example using the decision tool

We illustrate the use of the decision tool with a hypothetical example in which all of the 13 surveillance improvement strategies are implemented over the course of a year in a typical developing country setting. As described above, this is a common interval for assessing public health program progress. Because there are very scant data on specific country influenza surveillance systems and we could not identify quantitative global planning assumptions, e.g., from WHO, we inputted estimated values for probability and time which are based on relevant documentation, when available (e.g., planning assumptions from the U.S. *National Strategy for Pandemic Influenza Implementation Plan*, data from U.S. sentinel surveillance systems, U.S. data on numbers of physicians and hospitals, current times required for laboratory testing), and on the views of the study team regarding plausible estimates for a developing country setting. The first set of input values represents hypothetical 'baseline' conditions; the second reflects projected 'follow-up' values that might be obtained if all 13 improvement strategies are implemented over 12 months with projected quantitative targets for each step in the process model. The tool can be used both to strategically plan for implementation and to document achievements.

**Figure 2:**
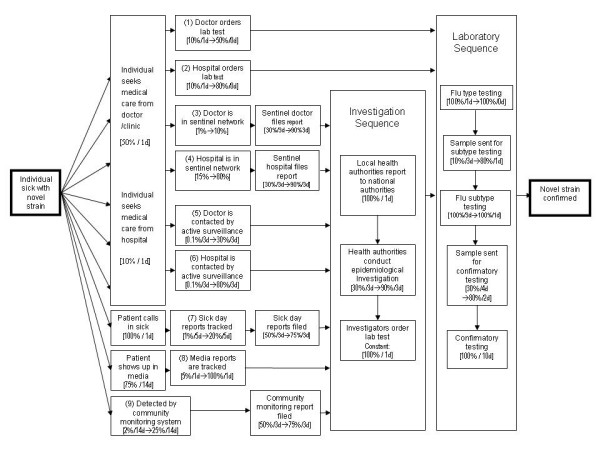
Illustrative example of the decision tool: Input values for the likelihood (probability) and timeliness (in days) of each step in the process model, for baseline and one-year follow up.

Figure 2 demonstrates the use of the tool by depicting our hypothetical *input values *for the baseline (current system) and follow-up (post improvement) periods for each part of the surveillance sequence. In this example, multiple improvement strategies are implemented concurrently – as reflected by changes between baseline and follow-up input values for steps in each detection path – i.e., more doctors order diagnostic laboratory testing, more doctors and hospitals are added to the sentinel surveillance network, active surveillance is implemented, worker sick days and electronic media are better tracked, community-based surveillance is implemented, reporting compliance from all sources increases, epidemiologic infrastructure is strengthened so that investigation of rumors is more likely, specimen transport is expedited, and laboratory infrastructure is strengthened so that testing is more likely and quicker. The tool then calculates the net probabilities and total time for the nine detection paths, i.e., to reach the final laboratory confirmation step through each pathway.

For purposes of demonstrating the model, we made the following assumptions about baseline and follow-up input values for selected steps in the model:

• We held constant at 50% the probability that the sick individual will seek medical care from a doctor (4), and at 10% the probability of seeking care from a hospital.

• We also held constant the delay in seeking care from a doctor or hospital, since we do not propose a strategy to increase patient demand for services (we used a low estimate of a 1-day delay in seeking medical care, but this can be held constant at a higher level, which would result in that additional number of days through each of the relevant detection paths).

• Since we wished to examine the strategy related to streamlined timing of notification rather than the level of reporting compliance from local to national public health authorities, we held constant at 100% the probability of reporting by local authorities to national authorities.

• Since such investigations routinely include laboratory testing, we held constant at 100% the probability of ordering of lab tests as part of case or outbreak investigations.

• Finally, since we handled the variability in probabilities for laboratory testing by varying the probability that a specimen is sent to each laboratory, we also held constant at 100% the probability that samples received by laboratories are tested.

We also used the same baseline and follow-up values for compliance in reporting by all local reporting sources, i.e., the percent of designated reporting sites that submit surveillance reports at a pre-defined "regular" interval:

• The probability of compliance is projected to increase from 50% at baseline to 75% at follow-up for community reporting sites and from 30% to 90% for clinical reporting sites

• We assume a constant 3-day delay in reporting from all sites, reflecting the average delay within a weekly reporting system.

The tool calculates the probability and time required for each of the nine detection paths, under the two scenarios (baseline and follow up). Table 2 shows the net *output values *calculated by the tool for our hypothetical example, and the resulting increases in probability and decreases in time required for each detection path between baseline and projected follow up.

**Table 2:** Comparison of influenza surveillance improvement strategies: Baseline and follow-up output values for hypothetical example

		Baseline		Follow-up	
		
		Probability (%)	Time (days)	Probability (%)	Time (days)
*Detection from clinical sites*					

Path 1	Doctor orders laboratory test	0.15	23	16.0	15
Path 2	Hospital orders laboratory test	0.03	23	5.1	15
Path 3	Reporting by sentinel doctor	0.0	30	2.6	23
Path 4	Reporting by sentinel hospital	0.0	30	4.2	23
Path 5	Active surveillance of doctors	0.0	30	8.6	23
Path 6	Active surveillance of hospitals	0.0	30	4.6	23

*Detection from community sites*					

Path 7	Tracking of employee sick days	0.0	35	8.6	28
Path 8	Tracking of electronic media	0.03	41	43.2	34
Path 9	Detection by community-based monitoring system	0.01	43	10.8	36

To provide a better understanding of how the tool works, we describe the process for three of those paths in greater detail.

#### Path 1: Doctor orders laboratory test

Based on the steps and input values shown in Figure 2, at baseline this requires an individual to:

• seek medical care (50% probability, 1 day delay),

• then for the doctor to order a diagnostic laboratory test (10% probability and 1 day delay),

• then for the laboratory sequence to unfold – sample is tested for influenza type (100% probability, 1 day),

• then it is sent to the next laboratory for subtype testing (10% probability, 3 day delay),

• the subtype testing is undertaken (100% probability, 3 day delay),

• then the sample is sent for confirmatory testing (30% probability, 4 day delay), and

• the confirmatory testing is completed (100% probability, 10 day delay).

The product of the sequential probabilities is: 0.5 × 0.1 × [laboratory sequence: 1 × 0.1 × 1 × 0.3 × 1] = 0.0015, or 0.15%, as reflected in Table 2. The total time for this path at baseline is the sum of the delays or time required at each step: 1 + 1 + [laboratory sequence: 1 + 3 + 3 + 4 + 10] = 23, also as reflected in Table 2.

For the follow-up scenario, we assume:

• Interventions to sensitize doctors to order diagnostic laboratory tests result in an increase by 5-fold in the percentage ordering such tests (input values increase from 10% to 50%).

• Interventions to strengthen the epidemiology and laboratory infrastructure result in an increase in the probability that samples are tested and a decrease in the time needed to perform the testing.

As a result, the sequential probabilities are: 0.5 × 0.5 × [laboratory sequence: 1 × 0.8 × 1 × 0.8 × 1] = 0.16, or 16%; and the total time is 1 + 0 [same day] + [laboratory sequence: 0 + 1 + 1 + 2 + 10] = 15. This represents an increase from 0.15% to 16% in the probability of detecting the case, and a reduction in time from 23 to 15 days.

#### Path 3: Doctor is in sentinel network

Here, the requirements at baseline are for the individual to:

• seek medical care from a doctor (50% probability, 1 day delay),

• for the doctor to be part of the country's sentinel surveillance system (1% probability),

• for the doctor to file the routine surveillance report (30% probability, 3 day delay),

• for the investigation sequence to unfold, including an epidemiologic investigation (30% probability, 3 day delay), and then for the laboratory sequence to unfold as described above.

The serial probabilities are: 0.5 × 0.01 × 0.3 × [investigation sequence: 1 × 0.3 × 1] × [laboratory sequence: 1 × 0.1 × 1 × 0.3 × 1] = 0.00001, or 0.001%; the total time required at baseline is: 1 + 3 + [1 + 3 + 1] + [1 + 3 + 3 + 4 + 10] = 30.

At follow up, after interventions to increase by 10-fold the percentage of doctors in the sentinel surveillance network (from 1% to 10%), improve reporting compliance 3-fold (from 30% to 90%), and improve the epidemiology and laboratory infrastructure, the probability of detection increases to only 2.6%.

#### Path 8: Tracking of electronic media

This path depends on information about a case or cluster in the community appearing in any electronic media or communications that can be tracked using electronic data mining methods such as the GPHIN system described below. Here, the main change between baseline and follow up is a hypothesized 20-fold increase, from 5% to 100%, that media reports are tracked. The serial probabilities of detection at baseline are: 0.75 × 0.05 × [investigation sequence 0.3] × [laboratory sequence: 0.03] = 0.0003, or 0.03%; and at follow up: 0.75 × 1 × [investigation sequence 0.9] × [laboratory sequence 0.64] = 0.43, or 43%. The savings in time, from 41 to 34 total days, comes exclusively from the 7 days reduced for the laboratory sequence.

These three examples highlight a number of important observations. First, sensitizing doctors to order diagnostic tests on suspicious patients is an efficient pathway in terms of assuring that the first doctor seen orders a test and also in bypassing the investigation sequence, but this path still depends on the individual seeking medical care. Second, as shown in the Path 3 example above, traditional sentinel surveillance has a number of important "filters" that together can impede the probability of case detection and confirmation. Third, a community-based strategy such as electronic data mining is not subject to the "filter" of the sick individual seeking care from and being reported by a doctor or hospital. Indeed, any pathway that relies on a sick individual to report to a doctor or hospital for medical care and subsequent reporting is subject to a substantial early "filter" – U.S. national planning assumptions are based on only 50% of such individuals seeking medical care, and this figure is likely much lower in developing countries. Thus, this step alone greatly reduces the probability of detection. Seeking to increase this health seeking behavior may overwhelm clinical facilities and may or may not result in significant improvements in likelihood of detection. Finally, as shown in Figure 2 and Table 2, reporting compliance for any system is also a potentially significant "filter", but potentially one that can be addressed through targeted program efforts. In this example, we projected a three-fold increase in compliance from sentinel providers (from 30% to 90%), and a 1.5-fold increase (from 50% to 75%) from community sources. The net probabilities of detection thus increase by this magnitude based on this factor alone.

Examination of the logic model and outputs for all paths in our illustrative example of the tool thus suggests a number of potentially valuable strategies to improve influenza surveillance:

• *Laboratory *– Because all nine detection paths include the Laboratory Sequence, slow execution of this sequence or failure of a sample to be accurately tested jeopardizes the effectiveness of all the preceding paths. Therefore, implementation of the three strategies related to improving laboratory diagnosis (improving laboratory diagnosis through training, equipping and proficiency testing; expediting specimen transport; and widely deploying rapid diagnostic tests) will simultaneously improve the probability and timeliness of case detection for all nine paths.

• *Epidemiology *– The Investigation Sequence also stems from a number of detection paths (3 through 9); therefore, the strategy to improve reporting compliance and the two strategies specifically related to the Investigation Sequence (improving epidemiologic capacity through training in applied epidemiology; streamlining notification, analysis and reporting through use of electronic and other appropriate technologies) are all critical to increase the probability and timeliness of case detection arising from multiple detection paths.

• *Tracking electronic media *– Surveillance methods that do not require the sick individual to seek medical care avoid this important "failure point" associated with the first six paths in our model, which reflect traditional surveillance systems. The use of data mining methods to track the vast amount of electronic media with potentially valuable information about the occurrence of suspicious disease in a community greatly improves surveillance coverage and timeliness. It is easy, quick, and inexpensive to implement because at least one such system currently exists (GPHIN) and can be enhanced with add-ons, e.g., to expand the range of foreign languages tracked [15].

• *Targeted laboratory testing *– The first six paths in our model stem from a sick individual seeking medical care from a doctor/clinic or hospital. Targeted laboratory testing by the initial health care provider involves widely disseminating guidelines to sensitize clinical providers to order influenza diagnostic laboratory tests for appropriate clinical cases [11,12]. This strategy is easier to implement widely and, in our hypothetical example, produces better probability and timeliness of case detection compared to alternative strategies related to clinical visits, i.e., sentinel reporting or active surveillance. Also, since there are fewer hospitals than physicians and sicker patients are more likely to go to a hospital, perhaps the first priority should be to sensitize the clinicians at all hospitals to appropriate guidelines for ordering laboratory testing for suspected cases of novel influenza.

• *Community-based monitoring *– Perhaps the most difficult pathway, at least in terms of public health implementation, is case detection through community-based monitoring systems. Establishing such systems is labor intensive and will require extensive mobilization and education of communities throughout a country and will also take more time (probably measured in years) than some of the other interventions [13]. Once established throughout a country, however, community-based monitoring systems can provide more comprehensive surveillance coverage for influenza, as well as for other diseases and conditions. Moreover, WHO has described the limitations of surveillance in resource-poor settings and commented that "Mobilizing communities to report unusual health events ... is the simplest form of cluster surveillance" [17].

### Seeking partnerships to improve surveillance systems

Government health authorities can implement the 13 potential surveillance improvement strategies on their own, drawing upon their resources as well as guidance from WHO. However, working with strategic partners outside their own system provides important opportunities to leverage partnerships to further improve the coverage, quality and timeliness of their influenza surveillance system and thereby further increase the probability and timeliness of case detection. Specific opportunities for such strategic partnerships are described below.

Strategies to improve surveillance coverage are inherently oriented toward the local level. Therefore, organizations whose activities are primarily at the grassroots or local level might be particularly well suited as strategic partners for national planners to help improve surveillance coverage. Failure to leverage such partners can mean greater effort and resources required on the part of national planners, lower levels of coverage at a given time, or delays in achieving broader coverage. Examples of potential partners include development agencies, nongovernmental organizations (NGOs), and even local businesses.

Strategies to improve surveillance quality include building laboratory and epidemiology capacity and targeted laboratory testing. Technical agencies with laboratory and/or epidemiology capacity are of potential strategic value to help improve international surveillance quality. Examples include academic institutions, Ministries of Health from other countries, development agencies, technically-oriented NGOs, international laboratory networks, and the Training in Epidemiology and Public Health Network. For example, the U.S. Department of Health and Human Services now has a formal agreement with the Institut Pasteur to partner on improving laboratory capacity in selected countries, beginning in Southeast Asia [18].

Strategies to improve the timeliness of surveillance include expedited transport of laboratory specimens within countries and from countries to reference laboratories, streamlined notification and analysis, and widespread deployment of rapid diagnostic tests. Technologically oriented businesses, academic institutions, and technical government agencies might be well suited strategic partners in this core surveillance area.

## Discussion

WHO has noted that "One of the most important functions of surveillance is to ensure the detection of unusual clusters of cases and of the occurrence of human-to-human transmission at the earliest possible stage, when public health interventions have the greatest chance to prevent or delay further national and international spread" [17]. Our study focused on ways to better assure early detection of such cases. We systematically identified possible strategies to improve coverage, quality and timeliness of global influenza case detection. We developed a process model onto which we mapped the strategies, built in quantitative measures of probability and time and programmed the model to allow users to vary input values for these measures and to calculate serial probabilities and total time for each detection path. This is the decision tool that permits comparison of different paths for case detection and different strategies to improve specific surveillance system processes. Examination of the process model and outputs from the tool revealed some strategies that are consistent with traditional public health practice and others that represent more novel approaches. These suggest that traditional sentinel surveillance is not designed to reliably detect early cases of a new disease in the community, and point instead to other detection paths that may be more suitable for pre-pandemic surveillance, especially community-based approaches. However, traditional efforts to build epidemiology and laboratory capacity are worthwhile, including more focused efforts within these, e.g., targeted laboratory testing through sensitization of doctors; expedited specimen transport; use of technologies to streamline data flow; and improving reporting compliance. Our model and published field studies suggest that rapid diagnostic tests also appear promising for surveillance purposes [14,16]. Finally, while several previous studies have noted the importance of early warning systems for timely case detection [12,19,20] and have described different ways to use real time electronic data streams [10,12,21,22], the robust use of electronic data mining for surveillance early warning remains a newer approach that has not yet reached its full potential for public health.

This study has both limitations and strengths that should be recognized. First, in our hypothetical example to illustrate application of the decision tool, we focused on potential improvements to surveillance systems. We acknowledge that some changes over time can weaken surveillance systems, e.g., due to economic decline or major disaster. Second, in the absence of identifiable global guidance, e.g., quantitative planning assumptions from WHO, or a suitable evidence base from a specific developing country, we relied on planning assumptions largely from the United States and the judgments of the study team to generate the hypothetical input values used to illustrate the decision tool. Thus, the outputs suggest potential rather than evidence-based benefits of the various surveillance approaches examined. The strengths of the study include our systematic approach to consider a broad range of potential improvement strategies, including traditional facility-based and newer community-based surveillance approaches and specific elements within these; and the development of a process model and decision tool that permit quantitative comparisons of alternative improvement strategies – improvements to specific elements in the surveillance process – implemented singly or in combination. Our novel approach can guide influenza surveillance programming in new and practical ways in countries around the world. This type of approach also can be applied to help improve surveillance for other diseases.

## Conclusion

Decision makers can apply the tool described here to their own programs – using baseline measures (or estimates) and planning targets – to assess the relative merits of and realistic expectations from implementation of the different surveillance improvement strategies, and thus select the ones that are most promising in their own context. This information can inform program policy and guide strategic investments to improve country surveillance systems within the larger context of global pandemic preparedness.

## Competing interests

The authors declare that they have no competing interests.

## Authors' contributions

MM conceived and led the study, developed the conceptual framework, helped design the model and interactive tool, and drafted and revised the manuscript. EC helped develop the conceptual framework, developed the process model and decision tool and helped to draft and review the manuscript. NL participated in the design of the study, development of the conceptual framework, and review of the manuscript. AGS, DMV and JAZ helped develop the conceptual framework, collect and analyze information, and review the manuscript. All authors read and approved the final manuscript.

## Pre-publication history

The pre-publication history for this paper can be accessed here:



## Supplementary Material

Additional file 1 Excel file with four worksheets: (1) "Inputs" – allows user to vary input values for all variable parameters in the decision tool for both baseline and follow-up time periods, (2) "Outputs" – displays a composite summary table of output calculations, followed by a summary for each detection path that contains both input values and the calculated output values, (3) "Graphs" – provides graphic display of output values for each path, comparing baseline to follow up, (4) "Notes" – provides additional notes for users. Click here for file
